# Association of fibroblast growth factor (FGF-21) as a biomarker with primary mitochondrial disorders, but not with secondary mitochondrial disorders (Friedreich Ataxia)

**DOI:** 10.1007/s11033-013-2767-0

**Published:** 2013-09-28

**Authors:** Mohammad Hossein Salehi, Behnam Kamalidehghan, Massoud Houshmand, Omid Aryani, Majid Sadeghizadeh, Mir Majid Mossalaeie

**Affiliations:** 1 Molecular Genetics Department, Tarbiat Modares University, Tehran, Iran; 2 Medical Genetics Department, National Institute for Genetic Engineering and Biotechnology (NIGEB), Tehran, Iran; 3 Pharmacy Department, Faculty of Medicine, University of Malaya (UM), 50603 Kuala Lumpur, Malaysia; 4 Medical Genetics Department, Special Medical Center, Tehran, Iran; 5 Parseh Medical Laboratory, Tehran, Iran

**Keywords:** Fibroblast growth factor 21 (FGF-21), Respiratory chain deficiency (RCD), Mitochondrial disorders (MIDs), Friedreich ataxia (FA), OXPHOS

## Abstract

Mitochondrial respiratory chain deficiencies are a group of more than 100 disorders of adults and children, with highly variable phenotypes. The high prevalence of mitochondrial disorders (MIDs) urges the clinician to diagnose these disorders accurately, which is difficult in the light of highly variable and overlapping phenotypes, transmission patterns and molecular backgrounds. Fibroblast growth factor 21 (FGF-21) is an important endocrine and paracrine regulator of metabolic homeostasis. The FGF-21 transcript is reported to be abundantly expressed in liver, but little is known about the regulation of FGF-21 expression in other tissues. FGF-21 could play a role in the metabolic alterations that are often associated with mitochondrial diseases. The aim of this study was to show the association of the FGF-21 biomarker with human primary MIDs and secondary MIDs in suspected patients in Iran. Serum FGF-21 levels were determined using ELISA in 47 mitochondrial patients, including 32 with primary MIDs, 15 patients with Friedreich ataxia as a secondary MID and 30 control subjects. Serum FGF-21 levels were significantly higher in subjects with the primary MIDs (*p* < 0.05), compared to subjects without MIDs. However, serum FGF-21 levels did not show significant increase in subjects with FA as a secondary MID. There is an association between increasing concentrations of FGF-21 with mitochondrial diseases, suggesting FGF-21 as a biomarker for diagnosis of primary MIDs in humans. However, this biomarker is not appropriate for the diagnosis of FA.

## Introduction

The human fibroblast growth factor (FGF) family includes at least 22 members with diverse biological functions, such as cell growth, cell differentiation, and wound healing [1]. Recent data has shown that this family may play important roles in defining and regulating the functions of some endocrine-relevant tissues and organs, as well as modulating various metabolic processes [2].

FGF-21 has been identified as a potent metabolic regulator [3]. Although the physiological role of FGF-21 in humans is not understood, higher circulating levels are strongly associated with certain metabolic states, where fat is accumulated in the liver, triglycerides are high and HDL cholesterol is low (all features associated with hepatosteatosis and dyslipidemia) [4]. FGF-21 is a recently identified player in carbohydrate and lipid metabolism [5–7]. Mounting evidence from animal-based studies suggests FGF-21 as a potent metabolic regulator with multiple beneficial effects on obesity and diabetes [8, 9]. Serum levels of FGF-21, which has been suggested as a potential candidate for the treatment of diabetes, are increased in obesity. In addition, there is a positive association of serum FGF-21 levels with age, several parameters of adiposity including BMI, waist circumference, waist-to-hip ratio, fat percentage, insulin resistance, and adverse lipid profiles [10].

FGF-21 is detected in fat [11] and muscle [12], and the concept of FGF-21 as a novel hepatokine, adipokine and even a myokine were suggested. However, FGF-21 is also present in the exocrine pancreas and b-cells at drastically higher levels than in the liver, adipose tissue and muscle [13–15]. In skeletal muscles, FGF-21 protein expression is essentially comparable to that of fasted liver. Hepatic FGF-21 transcription is upregulated by fasting [16, 17], thus skeletal muscle can be an important source of FGF-21 [12].

In this study, we measured and investigated the correlation of FGF-21 concentrations in the sera of patients with primary and secondary MIDs to assess whether it is a feasible biomarker for human RCD.

## Materials and methods

Patients with mitochondrial diseases were excluded by the fact that these defects were the result of primary MIDs. The genetic defect was established in mtDNA extracted from muscle and blood using established techniques. Confirmation of the mtDNA defect in each patient was sought but was not required for inclusion, assuming the clinical features were consistent and a pathogenic mutation had been confirmed within the pedigree. This approach was validated by a near-100 % rate of positive genetic tests where samples were available.

The suspected mitochondrial diseases were referred to the Medical Genetics Department at the Special Medical Center in Iran for investigation. Those with pathogenic mtDNA mutations were identified. Some FA patients were recruited as candidates for secondary MIDs because of the link between FA and MID. A total of 30 control subjects with no family history of MIDs were randomly selected.

Human FGF-21 enzyme-linked immunosorbent assay (ELISA) kits were obtained from BioVendor Laboratory Medicine (Modrice, Czech Republic). For the measurement of FGF-21. Serum samples were diluted 1:3 before the assay. Then, 100 μl of diluted sera, calibrators and quality controls were added to 96-well microtiter plates coated with an affinity-purified polyclonal anti-human FGF-21 antibody. The assay was conducted according to the manufacturer’s protocol.

A calibration curve was constructed by plotting the absorbance values at 450 nm versus the FGF-21 concentrations of the calibrators, and concentrations of unknown samples were determined using this calibration curve.

This assay was then used to measure serum FGF-21 levels in 47 Iranian mitochondrial disorders. In our study, 32 patients had primary MIDs and 15 patients had secondary MIDs (patients with FA). Our primary MID patients showed a pathogenic mutation or an abnormally low RC enzyme activity in muscle and had mitochondrial encephalomyopathy, lactic acidosis, myoclonus, epilepsy, ragged-red fibers mutation, point mutation, mtDNA deletions and mtDNA depletion.

## Results

The mean concentrations of FGF-21 in serum are 70 pg/ml (range 15–309 pg/ml) in healthy adults and 114 pg/ml (42–244 pg/ml) in healthy children, respectively [18]. Interestingly, patients with primary MIDs [346.2 pg/ml (17.5–1707); *n* = 32] had significantly higher serum FGF-21 levels than the controls [87.1 pg/ml (32–269.1); *n* = 30] (Table 1). No significant difference in serum FGF-21 levels was observed between FA patients and controls. [FA patients (*n* = 15), 71.6 pg/ml (23.9–93.4) vs. controls [87.1 pg/ml (32–269.1); *n* = 30] (Table 1).

**Table 1:** Correlations of serum FGF-21 levels with primary MIDs and FA

Group	Control	Primary MIDs	FA
Number of subjects	30	32	15
Gender (M/F)	17/13	17/15	9/6
FGF-21 level in male (pg/ml)	86.6	261.4	69.2
FGF-21 level in female (pg/ml)	88.7	422.4	75.1
FGF-21 level in all (pg/ml)	87.1	346.2	71.6

There were gender differences in serum FGF-21 levels [men (*n* = 17), median 261.4 pg/ml (interquartile range 17.5–1707] vs. women (*n* = 15), 442.4 pg/ml (25.2–1,599.1) among patients with primary MIDs, and there were no gender differences in serum FGF-21 levels [men (*n* = 9), median 69.2 pg/ml (interquartile range 23.9–81.8) vs. women (*n* = 6), 75.1 pg/ml (36.9–93.4) in FA patients] (Table 1).

## Discussion

Mitochondria are vital components of all nucleated cells because of the presence of the respiratory chain (RC), and are the major sites of energy production. Given the complexity of RC genetic inheritance and its function and regulation, a proper oxidative phosphorylation system (OXPHOS) requires the full assembly of functional proteins. Mutations of the nDNA and mtDNA genes encoding the different RC sub-complexes and their regulatory factors can produce a wide range of OXPHOS diseases with extremely heterogeneous clinical manifestations [19]. RC disorders are one of the most common causes of inherited metabolic disorders [20]. Over 100 genes govern the process of oxidative phosphorylation, and mutations in any of these can cause a mitochondrial respiratory chain defect [21].

It has become clear that OXPHOS may be impaired by mutations in many mitochondrial and even non-mitochondrial proteins, or may be disturbed as a secondary effect of other biochemical defects of intracellular metabolism [22]. OXPHOS defects caused by genes encoding non-OXPHOS mitochondrial proteins, such as frataxin, are responsible for FA [23]. FA is an autosomal recessive neurodegenerative disease characterized by progressive ataxia, neuropathy, skeletal abnormalities and cardiomyopathy. In FA, mutations in the nuclear gene encoding frataxin, which is involved in iron homeostasis in mitochondria, result in severe deficiencies of iron–sulphur clusters containing complexes I–III and of the Krebs cycle enzyme aconitase [22]. Defects of intracellular metabolism, particularly excess free radical generation, including nitric oxide or peroxynitrite, may cause secondary damage to the respiratory chain [22]. Other recessive nuclear mutations are known to affect structural subunits or assembly of mitochondrial respiratory chain complexes, and only FA was included in our prevalence figures [23].

Clinical experience in mitochondrial diseases suggest that ~75 % of adult-onset mitochondrial diseases are a consequence of primary mtDNA mutations [23]. The clinical presentation of RCD is highly variable in severity, age of onset, and the combination of organ systems involved. The factors contributing to this variability are poorly understood. Consequently, the diagnosis can be challenging, and there are very limited means of objectively monitoring disease progression [24]. Genetic disorders with impaired OXPHOS are extremely heterogeneous, as their clinical presentation ranges from lesions of single tissues or specialized structures, such as the optic nerve in mitochondrial DNA-associated Leber’s hereditary optic neuropathy and in nuclear DNA-associated dominant optic atrophy, to more widespread pathologies, including myopathies, peripheral neuropathies, encephalomyopathies, cardiopathies, or complex multisystem [25].

Increased concentrations of organic acids (particularly lactic acid and TCA cycle intermediates) in blood, urine, cerebrospinal fluid, as well as in tissues, are frequently encountered in patients with OXPHOS deficiencies, especially in children and in those with the most severe biochemical defects [26]. Serum biomarkers for MIDs include lactate, pyruvate, amino acids, creatine kinase, and possibly serum creatine [18, 27]. The ratio of lactate to pyruvate is sometimes raised, especially in children and in adults with encephalomyopathies, but is frequently normal in patients with progressive myopathies. Creatine kinase concentrations in serum are occasionally increased in people with mitochondrial diseases, mostly in the disorders that cause muscle damage [18].

The sensitivity of FGF-21 in identifying mitochondrial disorders with muscle involvement compared to those for lactate, pyruvate, lactate to pyruvate ratio, and creatine kinase suggest that the measurement of FGF-21 in serum by ELISA would be a useful first-line test in patients with suspected RCDs [18]. Recent evidence has indicated that there is a direct association between daily physical activity and serum FGF-21 levels [28]. There is also a positive association between mitochondrial myopathy and increased FGF-21 levels in the sera of mice [4]. Single RC-deficient muscle fibers induce the expression of the metabolic regulator FGF-21, with increased levels also seen in mouse plasma. These results strongly suggest that FGF-21 induction is a muscle-specific response, up-regulated especially in RC-deficient fibers, and that FGF-21 expression increases upon the disease progression, suggesting that their induction is related to the pathogenesis [4].

Suomalainen [18] presented a major step forward in the investigative options for patients with mitochondrial RCDs. They have focused their research on finding a biomarker to identify mitochondrial disease. In a mouse model with mitochondrial myopathy, they found that FGF-21 was induced in muscles and that concentrations in the serum were raised [4]. FGF-21 in serum seems to be a sensitive and specific quantitative biomarker for muscle pathology in a wide range of mitochondrial disorders in adults and children, including deficiencies in one or more of the respiratory chain enzyme complexes in the skeletal muscle. FGF-21 concentrations in serum seemed to increase with increasing clinical severity of the mitochondrial disease and muscle pathology [18]. Tyynismaa and colleagues [4] detected a threefold increase of FGF-21 levels in Deletor mice plasma, compared to controls. These results raised an interesting possibility that FGF-21 could be secreted from the diseased muscle fibers upon RC deficiency. RCD of single or multiple complexes resulted in high concentrations of FGF-21 in serum, including complex I deficiency, which is the most common form [29]. Respiratory chain defects induce FGF-21 expression in mouse skeletal muscle, which leads to raised concentrations in serum [30]. Systemic availability of FGF-21 could play a role in the metabolic alterations that are often are associated with mitochondrial diseases [3].

## Conclusions

Our data showed that serum FGF-21 levels in patients with primary MIDs were significantly higher than in control subjects, but showed no significant difference between patients with FA (as a secondary MID) than control subjects. Among these primary MIDs, women had moderately increased FGF-21 concentrations in serum. These results support the role of FGF-21 as a key regulator of metabolism in humans, and suggest that serum FGF-21 levels are associated with mitochondrial diseases and can be potentially used as a biomarker for primary MIDs. Although FGF-21 concentrations in serum were useful to differentiate patients with primary MIDs from healthy controls, FGF-21 concentrations were unable to diagnose FA in patients. This study provides the first clinical demonstration of the associations of serum FGF-21 levels with both primary MIDs and FA in humans.
